# A graph model for genomic prediction in the context of a linear mixed model framework

**DOI:** 10.1002/tpg2.20522

**Published:** 2024-10-07

**Authors:** Osval A. Montesinos‐López, Gloria Isabel Huerta Prado, José Cricelio Montesinos‐López, Abelardo Montesinos‐López, José Crossa

**Affiliations:** ^1^ Facultad de Telemática Universidad de Colima Colima Mexico; ^2^ Independent Consultant; ^3^ Department of Public Health Sciences University of California Davis Davis California USA; ^4^ Centro Universitario de Ciencias Exactas e Ingenierías (CUCEI) Universidad de Guadalajara Guadalajara Mexico; ^5^ Department of Statistics and Operations Research, and Distinguish Scientist Fellowship Program King Saud University Riyah Saudi Arabia; ^6^ AgCenter Louisiana State University Baton Rouge Louisiana USA; ^7^ Colegio de Postgraduados Montecillos Mexico; ^8^ International Maize and Wheat Improvement Center (CIMMYT) Mexico‐Veracruz Mexico

## Abstract

Genomic selection is revolutionizing both plant and animal breeding, with its practical application depending critically on high prediction accuracy. In this study, we aimed to enhance prediction accuracy by exploring the use of graph models within a linear mixed model framework. Our investigation revealed that incorporating the graph constructed with line connections alone resulted in decreased prediction accuracy compared to conventional methods that consider only genotype effects. However, integrating both genotype effects and the graph structure led to slightly improved results over considering genotype effects alone. These findings were validated across 14 datasets commonly used in plant breeding research.

## Core Ideas

- Genomic selection (GS) hinges on achieving high prediction accuracy of unobserved cultivars.
- Various statistical and machine learning models have been extensively explored to enhance prediction accuracy by exploring different machine learning models. Traditional statistical models assume independence among data points.
- Graph models are machine learning methods that aim capturing complex relationships and structures inherent in diverse datasets.
- Graph models represent data as interconnected, allowing for an understanding of relationships and dependencies of graph models within a linear mixed model framework.

**Abbreviations**
APC: average Pearson's correlation
GBLUP: genomic best linear unbiased predictor
GS: genomic selection
KNN: *k*‐nearest neighbors
MAAPE: mean arctangent absolute percentage error
NRMSE: normalized root mean square error

## 1 INTRODUCTION

Genomic selection (GS) is a cornerstone of modern agriculture, offering unprecedented precision in breeding programs to address the needs of a rapidly growing global population. By harnessing genetic information, GS enables breeders to identify and select individuals with desirable traits more efficiently and accurately than traditional methods (Crossa et al., 2017). This technology accelerates the development of crops and livestock with enhanced yields, improved nutritional content, and increased resilience to environmental stresses such as pests, diseases, and climate change. With the world population projected to reach 9.7 billion by 2050, ensuring sustainable food production has become imperative (United Nations, Department of Economic & Social Affairs, Population Division, 2019). GS not only expedites the breeding process but also ensures the production of high‐quality, high‐yielding varieties tailored to meet the specific challenges of modern agriculture, thus playing a critical role in securing food for the growing population while minimizing the environmental footprint of farming practices (VanRaden, 2017).

The implementation of GS in plant breeding, despite its promise, faces several challenges. Gathering and managing vast amounts of genetic and trait data can be costly, and ensuring prediction accuracy is complex due to factors like genetic diversity and environmental variations. Additionally, many breeding programs lack the necessary resources and expertise for sophisticated computational analysis. However, prediction models are essential for the success of GS, guiding breeders in selecting the best plants for breeding new varieties (Montesinos‐López et al., 2022). Simplifying these processes and making them more accessible is crucial for realizing the full potential of GS in agriculture.

In the field of genomic predictions, various statistical and machine learning models have been extensively explored. Bayesian models, linear models, mixed models, random forest, support vector machines, gradient boosting machines, and deep learning are among the methodologies investigated. The comparative effectiveness of these models, however, remains a topic of ongoing research and debate.

To address this gap and expand the repertoire of genomic prediction models, we investigate the efficacy of graph models within a mixed model framework. Graph models, increasingly popular in machine learning and data analysis, excel at capturing the complex relationships and structures inherent in diverse datasets. Unlike traditional statistical models, which assume independence among data points, graph models represent data as interconnected nodes and edges, allowing for a nuanced understanding of relationships and dependencies. This makes them well‐suited for analyzing data from various domains such as social networks, biological networks, recommendation systems, and transportation networks, where entities and their interactions exhibit intricate patterns.

Moreover, graph models offer flexibility in modeling both structured and unstructured data, enabling the integration of heterogeneous information sources and domain knowledge into the analysis process. As datasets grow in size and complexity, the scalability and adaptability of graph models make them increasingly valuable tools for uncovering hidden patterns, making predictions, and extracting insights from large‐scale, interconnected data (Battiston et al., 2014; Goyal & Ferrara, 2018).

**Core Ideas**

- Genomic selection (GS) hinges on achieving high prediction accuracy of unobserved cultivars.
- Various statistical and machine learning models have been extensively explored to enhance prediction accuracy by exploring different machine learning models. Traditional statistical models assume independence among data points.
- Graph models are machine learning methods that aim capturing complex relationships and structures inherent in diverse datasets.
- Graph models represent data as interconnected, allowing for an understanding of relationships and dependencies of graph models within a linear mixed model framework.

Graph models hold significant promise for genomic prediction, particularly in leveraging genetic markers. Genetic marker data often exhibit complex relationships, with markers representing specific genetic loci that may interact with each other or be linked to phenotypic traits of interest. Graph models offer a versatile framework for representing these relationships, allowing researchers to construct networks where nodes represent genetic markers and edges denote various types of relationships, such as linkage disequilibrium or epistatic interactions. By integrating information from multiple genetic markers and their interactions, graph models enable more accurate predictions of phenotypic traits, including disease susceptibility, yield potential, and other agronomic characteristics.

Furthermore, graph‐based approaches facilitate the incorporation of additional genomic information, such as gene expression data or pathway annotations, into predictive models, enhancing their predictive power and interpretability. Overall, the ability of graph models to capture the complex relationships among genetic markers makes them a promising tool for advancing genomic prediction research and improving breeding outcomes in agriculture and medicine.

In this study, we investigate the utilization of graph models within linear frameworks to assess their efficacy and potential to enhance the predictive capabilities of traditional genomic prediction models. This inquiry is significant, as graph models offer a means to seamlessly integrate supplementary information—such as biological pathways, gene–gene interactions, and epigenetic modifications—into predictive models, thereby improving their accuracy and interpretability. Furthermore, the adaptability and proficiency of graph models in encapsulating the intricate complexities of genomic data render them a promising prospect for advancing genomic prediction research and improving breeding outcomes across diverse domains, including agriculture, medicine, and beyond.

## 2 MATERIALS AND METHODS

### 2.1 Bayesian GLUP model

To evaluate the predictive accuracy of the traits of interest, we employed the widely recognized Bayesian genomic best linear unbiased predictor (GBLUP) model as outlined by Montesinos‐López et al. (2022). This model, denoted as the conventional, C model, was utilized with the subsequent predictor.

(1) $$Y_{i}=\mu +g_{i}+\varepsilon_{i}$$

In the provided model, $Y_{i}$ denotes the continuous response variable measured in the *i*th line; μ represents a general intercept, $g_{i},$ i=1,…,n signify the random effects of genotypes distributed as $N(0,\sigma_{g}^{2}G)$; G denotes the genomic relationship matrix computed as suggested by VanRaden (2008); and $\varepsilon_{i}$ denotes the random error components in the model, assumed to be independent normal random variables with a mean of 0 and a variance of $\sigma^{2}$. The implementation of this model was executed using the R statistical software (R Core Team, 2024), employing the BGLR library developed by Pérez and De Los Campos (2014).

### 2.2 Graph model

We presume the existence of an underlying network arrangement that governs the connections among lines. This arrangement is depicted through a graph denoted as *G* = (*V*, *E*), where the set of vertices *V* = {1,…, *n*} and the set of edges *E* comprise the pairs of vertices linked within the graph. The *adjacency matrix* of this graph, represented as $A=[A_{i,i\prime }]$ with *i*, *i*´ ∈ *V*, serving as a concise depiction of the connectivity structure. Each element of this matrix indicates whether pairs of vertices are adjacent within the graph, with $A_{i,i}\neq 0$ only when the pair i,i′∈ *E*. Our objective is to enhance prediction accuracy by accounting for interference among neighboring units attributed to genotypes. The marker information was used to construct graphs with nodes as lines (genotypes).

These matrices, $A=[A_{i,i\prime }],$ can be viewed as the adjacency matrix of an association network, that is, one where nodes with a sufficient level of association between node attributes are connected. For constructing this $A=[A_{i,i\prime }]$ we used (a) pairwise Pearson correlations between genotypes to construct the graph and (b) and *k*‐nearest neighbors (KNN) using as distance matrix one minus the pairwise Pearson´s correlations between genotypes. Nevertheless, employing correlations directly frequently yields dense matrices. Consequently, inducing sparsity becomes imperative to mitigate computational expenses. To achieve this objective, we investigated two methods: thresholding and KNN.

#### 2.2.1 Thresholding

This method consists of the following steps:

1. Let ($x_{i1}$, $x_{i2}$,…, $x_{ip}$) and ($x_{i\prime 1}$, $x_{i\prime 2}$,…, $x_{i\prime p}$) be the marker score vectors for genotypes *i* and *i´*, respectively. Then, the genomic correlation between these two individuals (genotypes) was calculated by the following expression.
$$cor\;\left(i,i^{\prime }\right)=\frac{\sum_{j\;=\;1}^{p}\left(1-0.5\left|x_{ij}-x_{i\prime j}\right|\right)}{p}$$

where the marker score is coded as 0, 1, or 2 for the genotype of M1M1, M1M2, or M2M2. Here, M1M1 and M2M2 denote the two homogenous genotypes and M1M2 denotes the heterogenous genotype.

1. After computing all the $\left(\begin{array}{c} n\\ 2 \end{array}\right)\;=\;\frac{n(n\;-\;1)}{2}$ pairs of correlation between the *n*‐genotypes, we computed the quantile 0.65 of all this pairs of correlations and this value was used as threshold ($\tau_{c}\}$ to build the genotype connections of the matrix as
$$A_{\left\{i,i\prime \right\}}=\left\{\begin{array}{c} 1\;\mathrm{if}\;\mathrm{cor}\left(i,i^{\prime }\right)\geq \tau_{c}\;\\ 0\;\mathrm{if}\;\mathrm{cor}\left(i,i^{\prime }\right)<\tau_{c} \end{array}\right.$$

#### 2.2.2 *k*‐nearest neighbors

1. We computed the correlation between each pair of genotypes as done in step 1 of the thresholding method.
2. Then we computed a matrix of distance between genotypes as $d_{i,i\prime }=\;1\;-\;cor\left(i,i^{\prime }\right)$ for *i* = 1,2,…,*n* and *i*´ = 1,2,…,*n*. Then with this information, we built the matrix of distance as $D=[d_{i,i\prime }]$.
3. Next, using this distance matrix, D, as input was used the KNN method to identify the k=10 neighbors of each genotype. This was done in R with the following code get.knn(D, k = 10).
4. After saving the 10 neighbors of each genotype, the coordinates of the A matrix were computed as follows:
$$A_{\left\{i,i\prime \right\}}=\left\{\begin{array}{c} 1\;\;\mathrm{if}\;i\prime \;\mathrm{was}\;\mathrm{selected}\;\mathrm{as}\;\mathrm{neighbor}\;\mathrm{of}\;i\\ 0\;\mathrm{if}\;i\prime \;\mathrm{not}\;\mathrm{was}\;\mathrm{selected}\;\mathrm{as}\;\mathrm{neighbor}\;\mathrm{of}\;i \end{array}\right.$$

After computing the A matrix, the prediction models implemented were as follows:

(2) $$Y_{i}=\mu +\sum_{i\prime =1}^{n}A_{\left\{i,i\prime \right\}}\gamma_{\left(i,i\prime \right)}+\varepsilon_{i}=\mu +g_{Ai}+\varepsilon_{i}$$

The components of model (2) mirror those of model (1) with one exception: the adjacency matrix $\;A_{\left\{i,i\prime \right\}}$, which delineates connections between genotypes *i* and *i´* within the directed graph, and $\gamma_{\left(i,i\prime \right)}$ represents the network effect of genotype *i* applied to the connected unit *i´*, when a connection exists between the two genotypes specified by these indices (known as neighbor, indirect genotype effect, or interference effect). Conventionally, diagonal elements of the adjacency matrix are uniformly set to zero to prevent self‐loops. In addition, $g_{Ai}\;=\;\sum_{i\;\prime =\;1}^{n}A_{\left\{i,i\prime \right\}}\gamma_{\left(i,i\prime \right)}$ represents the total network effect of genotype *i* applied to all connections and by adopting independent normal prior distributions for each $\gamma_{\left(i,i\prime \right)}$ with mean zero and variance $\sigma_{g_{A}}^{2}$, this implied that $g_{A}=\left(g_{A1},\text{…},g_{An}\right)$ is distributed as $\;N(0,\sigma_{g_{A}}^{2}G_{A})$; where $G_{A}=AA^{T}$ and A denotes the adjacency matrix defined before. It is important to point out that under this reparameterization, the second right component of the predictor given in Equation (2) corresponds to a GBLUP model. Also combining predictors in Equations (1) and (2) resulted the predictor given in Equation (3) as follows:

(3) $$Y_{i}=\mu +g_{i}+g_{Ai}+\varepsilon_{i}$$

The components of model (3) are the same to those described by models (1) and (2). Due to the fact that two methods (Thresholding and KNN) are implemented to compute the $A\;=[A_{i,i\prime }]$ matrix, for this reason, in this paper, the five resulting models were implemented:

Conventional, C, model implemented with the GBLUP model given in Equation (1).

GM_KNN_P1 computed the $A=[A_{i,i\prime }]$ matrix with the KNN method and it was implemented with model given in Equation (2).

GM_KNN_P2 computed the $A=[A_{i,i\prime }]$ matrix with the KNN method and it was implemented with model given in Equation (3).

GM_T_P1 computed the $A=[A_{i,i\prime }]$ matrix with the thresholding method and it was implemented with model given in Equation (2).

GM_T_P2 computed the $A=[A_{i,i\prime }]$ matrix with the thresholding method and it was implemented with model given in Equation (3).

It is important to note that these five versions of graph models, having been reformatted as conventional GBLUP models, are implemented for new lines in the same manner as conventional GBLUP models. Using the available information from the training data, the required parameters are estimated, and these trained models are then used to make predictions for new data.

### 2.3 Datasets

A concise overview of the datasets utilized in this study is provided in Table 1.

**TABLE 1:** Data description. Number of lines and markers.

Data	No. of lines	No. of markers
Indica	327	16,383
Japonica	320	16,383
Groundnut	318	8268
Maize	722	54,113
Wheat_1	1301	78,606
Wheat_2	1403	78,606
Wheat_3	1403	78,606
Wheat_4	1388	78,606
Wheat_5	1398	78,606
Wheat_6	1277	78,606
EYT_1	776	2038
EYT_2	775	2038
EYT_3	964	2038
Disease	438	11617

### 2.4 Cross‐validation strategies

For the comparison of the proposed and conventional models, we used cross‐validation. We implemented 10 random partitions, and in each partition, we randomly allocated 20% of the data to the testing set and the remaining 80% to the training set. For each testing set, we computed the accuracy using the following metrics: average Pearson's correlation (APC), normalized root mean square error (NRMSE), and mean arctangent absolute percentage error (MAAPE) (Montesinos‐López et al., 2022). Additionally, we calculated the percentage of best lines captured in the top 20% of the testing set (Best20). The average of the 10 partitions was reported as the prediction performance for these metrics.

## 3 RESULTS

The outcomes are presented in six sections. Sections 1 through 5 examine the accuracy of the following datasets: Disease, EYT_1, EYT_2, EYT_3, and Groundnut, while section 6 examines the results across datasets. The remaining results to datasets: Indica, Japonica, Maize, Wheat_1, Wheat_2, Wheat_3, Wheat_4, Wheat_5, and Wheat_6 are given in Figures S1–S9 and Tables S1–S9.

### 3.1 Disease

**FIGURE 1:**
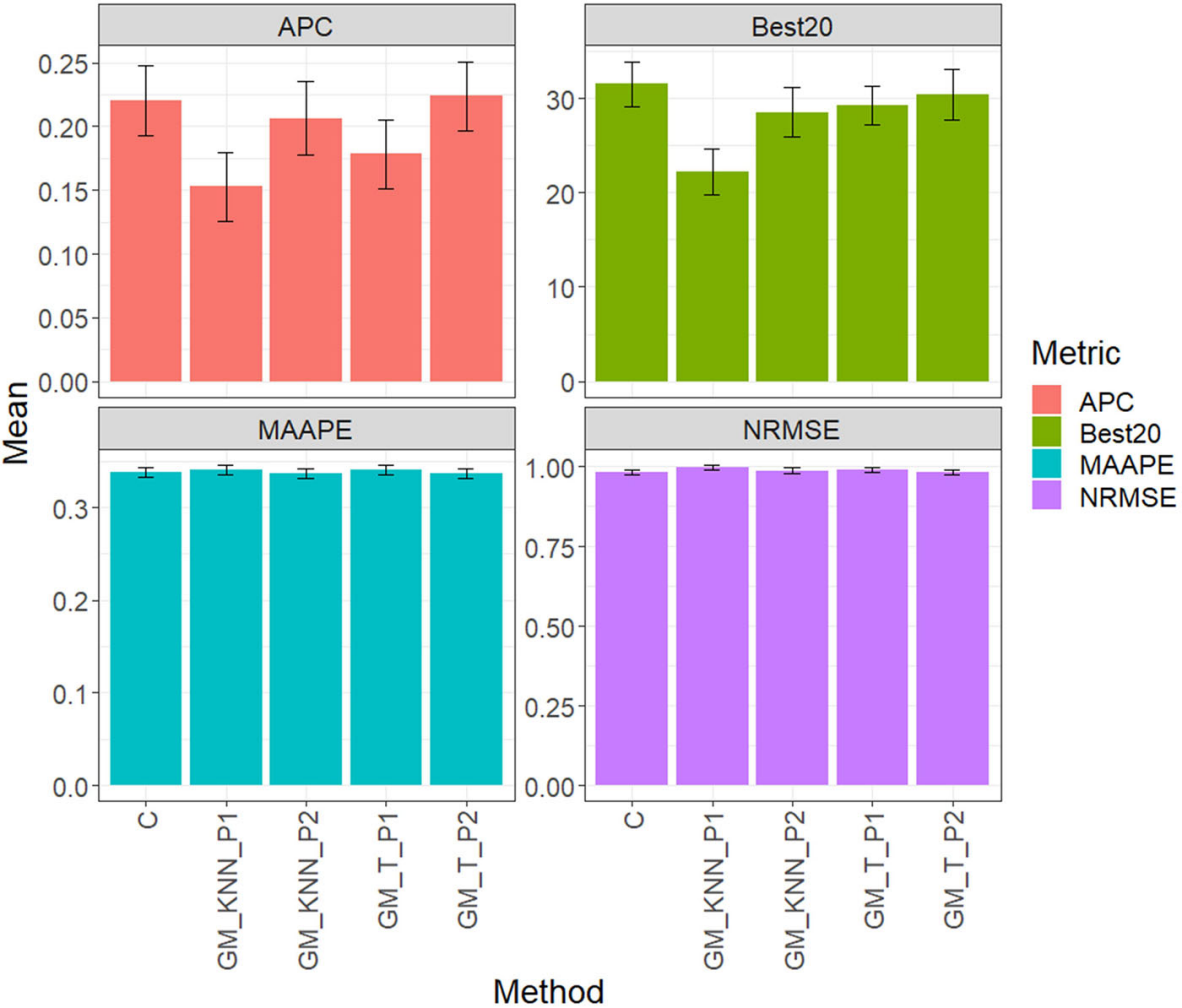
Disease dataset, comparison of prediction performance between the conventional method (C), GM_KNN_P1, GM_KNN_P2, GM_T_P1, and GM_T_P2 methods. The results are presented specifically for the genomic best linear unbiased predictor (GBLUP) model, with a detailed focus on four key metrics: (A) average Pearson's correlation (APC), (B) Best20, (C) mean arctangent absolute percentage error (MAAPE), and (D) normalized root mean square error (NRMSE).

Figure 1 and Table A1 present the evaluation results for the disease dataset, comparing five methods: conventional method (C), GM_KNN_P1, GM_KNN_P2, GM_T_P1, and GM_T_P2. The table includes APC, Best20, MAAPE, and NRMSE metrics, reflecting average performance across traits and environments.

For APC, GM_T_P2 led with an average of 0.2240, followed by C at 0.2203, and GM_KNN_P2 at 0.2063. GM_T_P1 averaged 0.1784, while GM_KNN_P1 had the lowest performance at 0.1527.

In Best20, C performed best with an average of 31.4815, followed by GM_T_P2 at 30.3704. However, C outperformed GM_T_P2 by 3.66%. GM_T_P1 averaged 29.2592, while GM_KNN_P1 had the weakest performance at 22.2222.

Regarding MAAPE, GM_KNN_P2 performed best with a score of 0.3366, followed by GM_T_P2 at 0.3367, slightly better than C at 0.3370. GM_T_P1 had the weakest performance at 0.3406.

In NRMSE, GM_T_P2 led with a score of 0.9809, followed by C at 0.9811, and GM_KNN_P2 at 0.9872. GM_T_P1 averaged 0.9905, slightly outperforming C. GM_KNN_P1 had the weakest NRMSE at 0.9974, showing notable differences compared to other methods. GM_T_P1 outperformed GM_KNN_P1 by 0.69%, GM_KNN_P2 by 1.03%, C by 1.64%, and GM_T_P2 by 1.65%.

### 3.2 EYT_1

**FIGURE 2:**
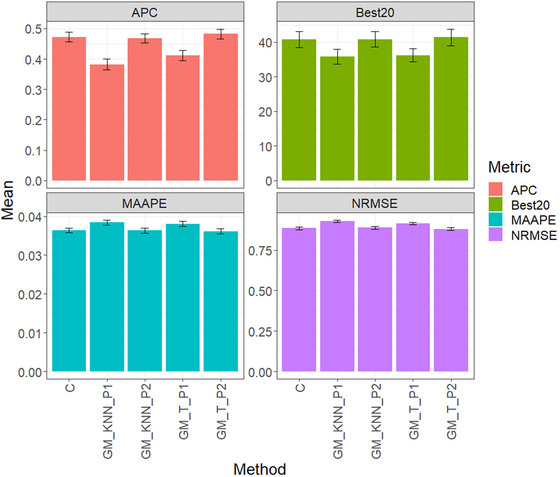
EYT_1 dataset, comparison of prediction performance between the conventional method (C), GM_KNN_P1, GM_KNN_P2, GM_T_P1, and GM_T_P2 methods. The results are presented specifically for the genomic best linear unbiased predictor (GBLUP) model, with a detailed focus on four key metrics: (A) average Pearson's correlation (APC), (B) Best20, (C) mean arctangent absolute percentage error (MAAPE), and (D) normalized root mean square error (NRMSE).

Figure 2 and Table A2 present the assessment findings for the EYT_1 dataset. GM_T_P2 emerges as the top performer in APC, averaging 0.4829, followed closely by the conventional method (C) at 0.4721 and GM_KNN_P2 at 0.4681. Notably, GM_T_P2 outperforms C by 2.23%, while GM_KNN_P2 shows a slight 0.86% decrease compared to C. GM_T_P1 averages 0.4113, whereas GM_KNN_P1 exhibits the least favorable performance at 0.3823.

For the Best20 metric, GM_T_P2 leads with an average of 41.4516, followed by GM_KNN_P2 at 40.8871. However, GM_T_P2 outperforms GM_KNN_P2 by 1.38%. C shows an average of 40.8065, slightly lower than GM_KNN_P2. GM_T_P1 records 36.2097, while GM_KNN_P1 displays the least favorable performance at 35.8065.

In terms of MAAPE, GM_T_P2 leads with a score of 0.0362, closely followed by GM_KNN_P2 with 0.0363, slightly ahead of C at 0.0364. GM_T_P2 outperforms GM_KNN_P2 by 0.48%. GM_T_P1 averages 0.0381, while GM_KNN_P1 has the least favorable performance at 0.0384.

In NRMSE evaluation, GM_T_P2 leads with a score of 0.8801, surpassing C at 0.8856 and GM_KNN_P2 at 0.8890. GM_T_P1 averages 0.9148, slightly outperforming C. Conversely, GM_KNN_P1 performs least favorably at 0.9280.

### 3.3 EYT_2

**FIGURE 3:**
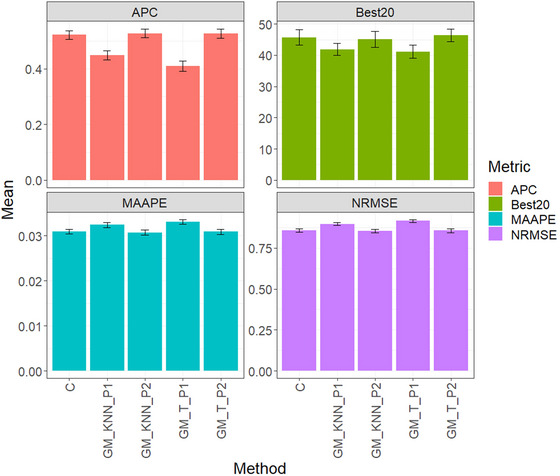
EYT_2 dataset, comparison of prediction performance between the conventional method (C), GM_KNN_P1, GM_KNN_P2, GM_T_P1, and GM_T_P2 methods. The results are presented specifically for the genomic best linear unbiased predictor (GBLUP) model, with a detailed focus on four key metrics: (A) average Pearson's correlation (APC), (B) Best20, (C) mean arctangent absolute percentage error (MAAPE), and (D) normalized root mean square error (NRMSE).

Figure 3 and Table A3 display the evaluation findings for the EYT_2 dataset. GM_KNN_P2 ranked highest in APC with an average of 0.5272, followed by GM_T_P2 at 0.5070, and C at 0.5220. Notably, GM_KNN_P1 showed distinct performance at 0.4483, while GM_T_P1 scored lowest at 0.4101, resulting in significant differences compared to other methods.

For the Best20 metric, GM_T_P2 stood out with an average of 46.4516, followed by C at 45.7258, and GM_KNN_P2 at 45.0807. Conversely, GM_T_P1 had the lowest performance at 41.1290, showing notable decreases compared to other models.

Concerning MAAPE, GM_KNN_P2 performed best with a score of 0.0307, followed closely by GM_T_P2 at 0.0309 and C at 0.0310. GM_KNN_P1 scored 0.0324, while GM_T_P1 had the least favorable performance at 0.0331, showing significant differences compared to other methods.

For NRMSE, GM_KNN_P2 achieved the highest score of 0.8539, followed by GM_T_P2 at 0.8553, and C at 0.8578. Conversely, GM_T_P1 had the lowest performance at 0.9145, showing significant differences compared to other methods.

### 3.4 EYT_3

**FIGURE 4:**
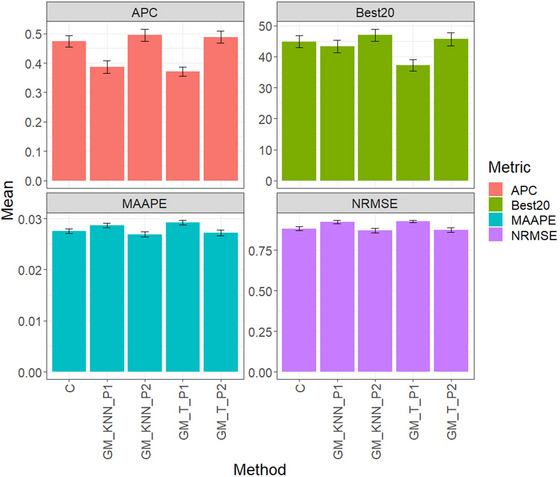
EYT_3 dataset, comparison of prediction performance between the conventional method (C), GM_KNN_P1, GM_KNN_P2, GM_T_P1, and GM_T_P2 methods. The results are presented specifically for the genomic best linear unbiased predictor (GBLUP) model, with a detailed focus on four key metrics: (A) average Pearson's correlation (APC), (B) Best20, (C) mean arctangent absolute percentage error (MAAPE), and (D) normalized root mean square error (NRMSE).

Figure 4 and Table A4 summarize the assessment results for the EYT_3 dataset. The top‐performing model, GM_KNN_P2, achieved an average APC score of 0.4951, outperforming GM_T_P2 by 1.45%. GM_T_P2 secured the second position with an average of 0.4880, while the conventional method (C) claimed third place with an average of 0.4737. GM_KNN_P1 demonstrated a distinct performance level with an average score of 0.3864, outperforming GM_T_P1 significantly, which had an average of 0.3707.

Regarding Best20, GM_KNN_P2 emerged as the leader with an average of 46.9872, surpassing GM_T_P2 by 2.95%. GM_T_P2 achieved an average of 45.6410, with C trailing slightly at 44.8718. GM_KNN_P1 had an average of 43.2693, while GM_T_P1 showed the least favorable performance with an average of 37.1795.

For metric MAAPE, GM_KNN_P2 showcased outstanding performance with a score of 0.0269, slightly surpassing GM_T_P2 by 1.10%. GM_T_P2 displayed a commendable average MAAPE of 0.0272, whereas C had an average MAAPE of 0.0276. GM_KNN_P1 recorded an average MAAPE value of 0.0287, while GM_T_P1 demonstrated the least favorable performance with a score of 0.0293.

Concerning metric NRMSE, GM_KNN_P2 dominated with a score of 0.8698, outperforming GM_T_P2 by 0.43%. GM_T_P2 showcased an average NRMSE of 0.8735, with C slightly behind at 0.8816. GM_KNN_P1 registered an average NRMSE value of 0.9220, while GM_T_P1 exhibited the least favorable performance with a score of 0.9267.

### 3.5 Groundnut

**FIGURE 5:**
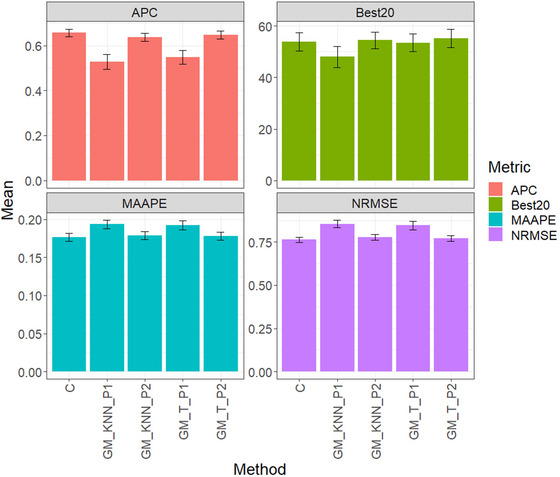
Groundnut dataset, comparison of prediction performance between the conventional method (C), GM_KNN_P1, GM_KNN_P2, GM_T_P1, and GM_T_P2 methods. The results are presented specifically for the genomic best linear unbiased predictor (GBLUP) model, with a detailed focus on four key metrics: (A) average Pearson's correlation (APC), (B) Best20, (C) mean arctangent absolute percentage error (MAAPE), and (D) normalized root mean square error (NRMSE).

Figure 5 and Table A5 show the evaluation results for the groundnut dataset. The APC metric indicates that model C scored highest with an average of 0.6567, followed closely by GM_T_P2 at 0.6476 and GM_KNN_P2 at 0.638. Despite C outperforming GM_T_P2 by 1.41%, GM_T_P1 achieved a distinct performance level with an average score of 0.5487. In contrast, GM_KNN_P1 had the least favorable performance at 0.527, resulting in significant decreases compared to other models.

For the Best20 metric, GM_T_P2 led with an average score of 55.1923, followed closely by GM_KNN_P2 at 54.423 and C at 53.8462. Despite GM_T_P2 outperforming GM_KNN_P2 by 1.41%, GM_T_P1 showed a distinct level of performance, registering an average score of 53.4615. Conversely, GM_KNN_P1 had the least favorable performance at 48.0769, resulting in notable decreases compared to other models.

In terms of MAAPE, C achieved a notable score of 0.1762, surpassing GM_T_P2 and GM_KNN_P2, while GM_T_P1 scored 0.1924. GM_KNN_P1 displayed the least favorable performance among the assessed methods.

Regarding NRMSE, C showcased outstanding performance with a noteworthy score of 0.763, followed closely by GM_T_P2 at 0.7709 and GM_KNN_P2 at 0.7776. GM_T_P1 had an average NRMSE value of 0.846, while GM_KNN_P1 displayed the least favorable performance with a score of 1, resulting in notable decreases compared to other models.

### 3.6 Across datasets

**FIGURE 6:**
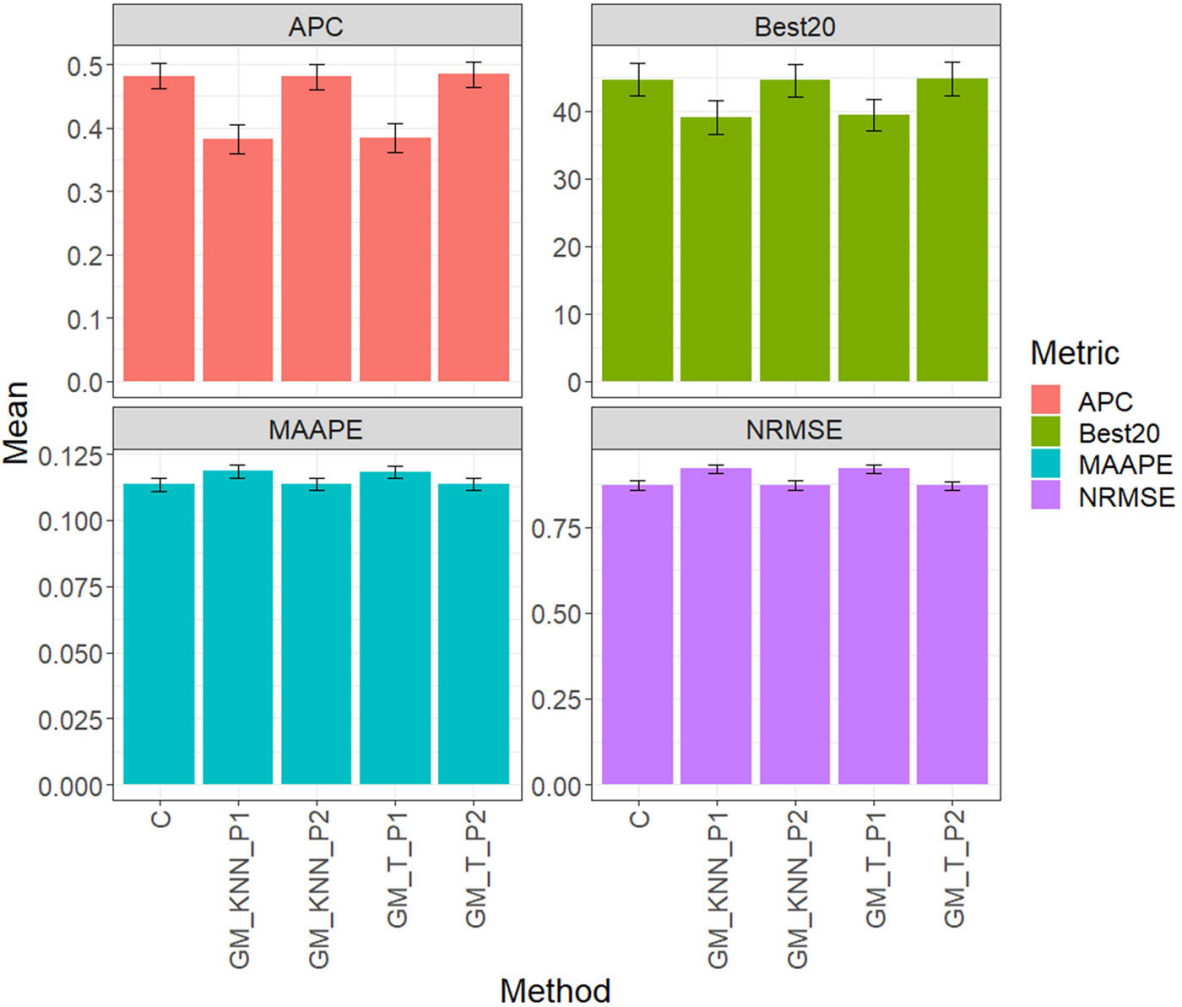
Across_datasets, comparison of prediction performance between the conventional method (C), GM_KNN_P1, GM_KNN_P2, GM_T_P1, and GM_T_P2 methods. The results are presented specifically for the genomic best linear unbiased predictor (GBLUP) model, with a detailed focus on four key metrics: (A) average Pearson's correlation (APC), (B) Best20, (C) mean arctangent absolute percentage error (MAAPE), and (D) normalized root mean square error (NRMSE).

In Figure 6 and Table A6, you will find the evaluation results across datasets. Model GM_T_P2 performed best according to the APC metric, achieving an average score of 0.485, closely followed by model C at 0.482 and model GM_KNN_P2 at 0.481. Conversely, GM_KNN_P1 exhibited the least favorable performance with an APC of 0.382, resulting in significant decreases compared to GM_T_P1, GM_KNN_P2, GM_T_P2, and model C.

For the Best20 metric, GM_T_P2 led with an average score of 44.792, followed by model C at 44.647 and model GM_KNN_P2 at 44.486. Conversely, GM_KNN_P1 showcased the least favorable performance with an average of 39.041, resulting in notable decreases compared to other models.

In terms of MAAPE, model C achieved a notable score of 0.113, surpassing GM_T_P2 and GM_KNN_P2, while GM_T_P1 performed least favorably with a score of 0.118, resulting in decreases compared to other models.

Regarding NRMSE, model GM_T_P2 showcased outstanding performance with a noteworthy score of 0.870, followed closely by model C at 0.871 and GM_KNN_P2 at 0.872. Conversely, GM_T_P1 performed least favorably with a score of 0.919, resulting in notable decreases compared to other models.

## 4 DISCUSSION

GS is embraced globally for its precision, speed, and cost‐effectiveness in breeding programs. By analyzing an organism's entire genome, breeders swiftly identify and select individuals with desired traits, leading to accelerated genetic improvement. This approach broadens genetic diversity, enhances resilience, and meets market demands more efficiently. GS optimizes resources, reduces breeding costs, and fosters sustainable agricultural practices. Its adoption signifies a transformative shift toward faster, more precise, and customized breeding strategies worldwide.

Many strategies are being under research to increase the efficiency of this prediction methodology. The success of statistical machine learning models used in genomic prediction has been notable, driving advancements in precision breeding and agricultural productivity. These models leverage vast genomic datasets to predict phenotypic (or breeding values) of traits, allowing breeders to select superior individuals more efficiently. Techniques like Ridge regression, mixed models, and Bayesian methods (Bayes A, Bayes B, Bayes Lasso, and random forest) have demonstrated efficacy in various breeding programs, contributing to accelerate genetic gains and the development of improved crop and livestock varieties. However, challenges remain, including the need for robust validation and calibration of models across diverse populations and environments. Despite these challenges, the continued refinement and integration of machine learning approaches hold great promise for further enhancing genomic prediction accuracy and applicability in agricultural breeding programs.

In this paper, we investigate the potential of graph models within the context of genomic prediction. Graph models serve as probabilistic modeling frameworks that illustrate the conditional relationships between random variables through a graph structure. This modeling technique encompasses a range of statistical and data mining methods, making it well‐suited for analyzing the collective distribution of a group of random variables. Originating from genetics (Wright, 1921) and applied across disciplines such as social sciences and economics (e.g., Haavelmo, 1943), graph models, rooted in path analysis, elucidate directed dependencies among variables. Path analysis, essentially a variant of multiple regression, emphasizes causal relationships and is represented in graph models by nodes representing variables interconnected by directed edges.

Graph models have emerged as indispensable tools across diverse research landscapes. Their widespread adoption in fields ranging from social sciences to genetics underscores their versatility and efficacy in analyzing complex data from observational studies. Graph models offer utility in modeling complex relationships within the genome due to their ability to capture intricate dependencies among genetic variables. In genomic contexts, where interactions between genes, environmental factors, and phenotypic traits are multifaceted, graph models provide a visual and analytical framework to represent and understand these relationships. By depicting directed edges between genetic elements, such as genes, SNPs (single nucleotide polymorphisms), or genomic regions, graph models can elucidate the causal pathways and regulatory networks underlying trait variation. Moreover, graph models accommodate the integration of diverse data types, including gene expression profiles, epigenetic modifications, and environmental variables, facilitating comprehensive analyses of genomic complexity. This flexibility enables researchers to uncover hidden associations, identify key regulatory factors, and ultimately enhance the predictive accuracy of genomic models. Therefore, graph models offer valuable insights into the intricate architecture of the genome, contributing to the advancement of precision breeding and personalized medicine.

Additionally, advancements in computational techniques have facilitated the integration of graph models with cutting‐edge technologies such as deep learning, enabling the exploration of intricate data structures, and uncovering latent patterns. As research continues to evolve, graph models stand poised to play an increasingly pivotal role in elucidating complex phenomena, driving innovation, and shaping the future of interdisciplinary inquiry.

### 4.1 Type of graph models

The choice between Bayesian and non‐Bayesian graph models depends on factors such as the available computational resources, the amount of available data, the desired level of interpretability, and the specific goals of the analysis. The connection between the variables constitutes the structure of the graph in a Bayesian graph model and is treated an uncertain and thus assigned a prior probability distribution that can be updated (based on the observed data) to obtain a posterior distribution. The advantage of Bayesian graph models is that they involve probabilistic inference over the graph structure. In non‐Bayesian graph models, the structure of the graph is considered fixed and determined based on predefined or optimized criteria. Non‐Bayesian graph models are computationally efficient and easier to interpret compared to their Bayesian counterpart.

### 4.2 Genomic prediction of graph models versus linear mixed models—Advantages

The integration of graph models with linear mixed models offers a powerful framework for genomic prediction in plant breeding by effectively capturing genetic relationships, incorporating genomic information, handling high‐dimensional data, accounting for population structure, and facilitating the integration of prior knowledge. Graph models allow for the integration of genomic data, such as SNPs into the prediction model. These molecular markers can be represented as nodes in the graph, with edges indicating relationships such as linkage disequilibrium or physical proximity. By combining genomic information with phenotypic data, linear mixed models can leverage the additional predictive power provided by genetic markers.

Graph models offer a flexible framework for representing and analyzing high‐dimensional data by capturing the underlying structure and dependencies among markers. Linear mixed models combined with graph‐based approaches can effectively handle high‐dimensional genomic data, leading to more efficient modeling process. Furthermore, graph models can represent population structures as networks of relatedness among individuals, allowing linear mixed models to account for population stratification and admixture effects and thus mitigating confounding effects and potentially improving the accuracy of genomic predictions. As already mentioned, graph models provide a framework for incorporating prior knowledge, such as known biological pathways or gene–gene interactions, into the prediction model. By encoding prior information as edges or constraints in the graph, linear mixed models can leverage domain expertise to improve prediction accuracy.

Our results from implementing graph models for genomic prediction demonstrate their versatility, as they can be integrated with conventional mixed models and Bayesian methods. Overall, the graph model proposed in this study did not outperform the conventional GBLUP method in terms of prediction accuracy. However, the worst performance was observed when only the graph structure was used in the predictor. Slightly better results were obtained when the conventional predictor of the GBLUP model was used together with to the graph structure. It is possible that a more exhaustive search for the hyperparameters used in constructing the graph structure (adjacency matrix) could enhance the prediction performance of the proposed graph model. Given that the adjacency matrix is specifically designed to reflect the suspected underlying connections among genotypes, its construction is of paramount importance. Therefore, to ensure the successful implementation of graph models, precision in building the adjacency matrix is crucial. Failure to properly specify or account for significant network structures could lead to biased estimations and poor prediction performance.

### 4.3 Genomic prediction of graph models versus linear mixed models—Disadvantages

Graph models may not always outperform GBLUP in genomic prediction for several reasons. If the genetic architecture is simple, with few large‐effect loci dominating the trait variation, simpler methods like GBLUP may perform as well as or even better than graph models. Graph models often introduce additional complexity compared to GBLUP. While this complexity can capture intricate genetic relationships and interactions, it may require larger sample sizes to estimate parameters accurately. In situations where data are limited, simpler models like GBLUP might be more robust and less prone to overfitting. In Bayesian graph models, inferring the structure of the graph (i.e., determining which genetic markers are connected) can be challenging, especially with high‐dimensional genomic data. For traits influenced by environmental factors or nongenetic sources of variation, the added complexity of graph models may not necessarily lead to improved prediction accuracy. In such cases, simpler models like GBLUP, which focus primarily on genetic relatedness, may suffice for genomic prediction.

Another disadvantage of integrating graph models with conventional GBLUP is the increased demand for computational resources. This is due to the need to estimate more parameters, which can significantly escalate the computational burden. The high dimensionality and complexity of these models require more memory and processing power, which can be a limiting factor, especially in large‐scale genomic studies.

Furthermore, Bayesian graph models present additional computational challenges. Inferring the structure of the graphs in Bayesian frameworks can be computationally intensive and complex. The process involves exploring a vast space of possible graph configurations to identify the most probable structure, which can be extremely demanding in terms of computation. This complexity can lead to longer processing times and the need for specialized computational techniques and resources to manage the high computational load efficiently.

Therefore, while graph models offer a promising approach for genomic prediction, addressing these computational challenges is essential for their practical implementation. Advances in computational methods and resources will be critical for harnessing the full potential of graph models in genomic studies.

## 5 CONCLUSIONS

In this research, we propose the use of graph models within Bayesian frameworks for genomic predictions. Our findings indicate that utilizing network effects alone in the predictor does not surpass the performance of the conventional GBLUP model across different datasets. Similarly, incorporating both genotype effects and network effects into the conventional GBLUP model did not yield significant improvements. We observed no substantial differences between the two methods used to construct the adjacency matrix. Based on our empirical evidence, we recommend further research on graph models in GS to investigate how to adapt the graph models to genomic prediction to potentially surpass conventional GBLUP models in prediction accuracy. Graph models hold promise for genomic prediction in plant breeding due to their ability to incorporate complex genetic relationships, efficiently use genomic data networks, and adapt to diverse data structures. However, more research is required to enhance current GS models. Their flexibility, scalability, and capability to leverage extensive genomic datasets make graph models attractive tools for accelerating genetic gain and improving breeding efficiency in plant improvement programs.

## AUTHOR CONTRIBUTIONS

**Osval A. Montesinos‐López**: Conceptualization; data curation; formal analysis; supervision; writing—original draft; writing—review and editing. **Gloria Isabel Huerta Prado**: Conceptualization; formal analysis; writing—original draft; writing—review and editing. **José Cricelio Montesinos‐López**: Conceptualization; formal analysis; investigation; software; writing—original draft; writing—review and editing. **Abelardo Montesinos‐López**: Conceptualization; formal analysis; investigation; methodology; writing—original draft; writing—review and editing. **José Crossa**: Conceptualization; methodology; writing—original draft; writing—review and editing.

## CONFLICT OF INTEREST STATEMENT

The authors declare no conflicts of interest.

## Supporting information



Supporting information

## Data Availability

The phenotypic and genomic data used in this study are available for download from the following link: https://github.com/osval78/BLUES_Across_LOC_Geno_Markers. There are not original data associated with this study.

## APPENDIX A

### A.1

**TABLE A1:** Comparison of prediction performance between methods C, GM_T_P1, GM_T_P2, GM_KNN_P1, and GM_KNN_P2 for the line selection in genetic improvement (dataset: disease) based on average Pearson's correlation (APC), Best20, mean arctangent absolute percentage error (MAAPE), and normalized root mean square error (NRMSE).

Dataset	Method	Metric	Mean	SE
Disease	C	APC	0.2203	0.0277
Disease	C	Best20	31.4815	2.4199
Disease	C	MAAPE	0.3374	0.0052
Disease	C	NRMSE	0.9811	0.0081
Disease	GM_KNN_P1	APC	0.1527	0.0267
Disease	GM_KNN_P1	Best20	22.2222	2.4720
Disease	GM_KNN_P1	MAAPE	0.3401	0.0054
Disease	GM_KNN_P1	NRMSE	0.9974	0.0077
Disease	GM_KNN_P2	APC	0.2063	0.0289
Disease	GM_KNN_P2	Best20	28.5185	2.6164
Disease	GM_KNN_P2	MAAPE	0.3366	0.0048
Disease	GM_KNN_P2	NRMSE	0.9872	0.0088
Disease	GM_T_P1	APC	0.1784	0.0268
Disease	GM_T_P1	Best20	29.2592	2.0586
Disease	GM_T_P1	MAAPE	0.3406	0.0051
Disease	GM_T_P1	NRMSE	0.9905	0.0066
Disease	GM_T_P2	APC	0.2240	0.0270
Disease	GM_T_P2	Best20	30.3704	2.6776
Disease	GM_T_P2	MAAPE	0.3367	0.0050
Disease	GM_T_P2	NRMSE	0.9809	0.0080

**TABLE A2:** Comparison of prediction performance between methods C, GMT_P1, GM_T_P2, GM_KNN_P1, and GM_KNN_P2 for the line selection in genetic improvement (dataset: EYT_1) based on average Pearson's correlation (APC), Best20, mean arctangent absolute percentage error (MAAPE), and normalized root mean square error (NRMSE).

Dataset	Method	Metric	Mean	SE
EYT_1	C	APC	0.4721	0.0159
EYT_1	C	Best20	40.8065	2.2444
EYT_1	C	MAAPE	0.0364	0.0006
EYT_1	C	NRMSE	0.8856	0.0090
EYT_1	GM_KNN_P1	APC	0.3823	0.0176
EYT_1	GM_KNN_P1	Best20	35.8065	2.1861
EYT_1	GM_KNN_P1	MAAPE	0.0384	0.0007
EYT_1	GM_KNN_P1	NRMSE	0.9280	0.0079
EYT_1	GM_KNN_P2	APC	0.4681	0.0155
EYT_1	GM_KNN_P2	Best20	40.8871	2.1722
EYT_1	GM_KNN_P2	MAAPE	0.0363	0.0006
EYT_1	GM_KNN_P2	NRMSE	0.8890	0.0092
EYT_1	GM_T_P1	APC	0.4113	0.0164
EYT_1	GM_T_P1	Best20	36.2097	1.9236
EYT_1	GM_T_P1	MAAPE	0.0381	0.0007
EYT_1	GM_T_P1	NRMSE	0.9148	0.0075
EYT_1	GM_T_P2	APC	0.4829	0.0161
EYT_1	GM_T_P2	Best20	41.4516	2.4125
EYT_1	GM_T_P2	MAAPE	0.0362	0.0006
EYT_1	GM_T_P2	NRMSE	0.8801	0.0094

**TABLE A3:** Comparison of prediction performance between methods C, GM_T_P1, GM_T_P2, GM_KNN_P1, and GM_KNN_P2 for the line selection in genetic improvement (dataset: EYT_2) based on average Pearson's correlation (APC), Best20, mean arctangent absolute percentage error (MAAPE), and normalized root mean square error (NRMSE).

Dataset	Method	Metric	Mean	SE
EYT_2	C	APC	0.5220	0.0154
EYT_2	C	Best20	45.7258	2.5403
EYT_2	C	MAAPE	0.0310	0.0005
EYT_2	C	NRMSE	0.8578	0.0104
EYT_2	GM_KNN_P1	APC	0.4483	0.0167
EYT_2	GM_KNN_P1	Best20	41.8549	1.9385
EYT_2	GM_KNN_P1	MAAPE	0.0324	0.0006
EYT_2	GM_KNN_P1	NRMSE	0.8964	0.0096
EYT_2	GM_KNN_P2	APC	0.5272	0.0158
EYT_2	GM_KNN_P2	Best20	45.0807	2.6205
EYT_2	GM_KNN_P2	MAAPE	0.0307	0.0006
EYT_2	GM_KNN_P2	NRMSE	0.8539	0.0113
EYT_2	GM_T_P1	APC	0.4101	0.0188
EYT_2	GM_T_P1	Best20	41.1290	2.0762
EYT_2	GM_T_P1	MAAPE	0.0331	0.0005
EYT_2	GM_T_P1	NRMSE	0.9145	0.0087
EYT_2	GM_T_P2	APC	0.5270	0.0168
EYT_2	GM_T_P2	Best20	46.4516	2.0306
EYT_2	GM_T_P2	MAAPE	0.0309	0.0005
EYT_2	GM_T_P2	NRMSE	0.8553	0.0113

**TABLE A4:** Comparison of prediction performance between methods C, GM_T_P1, GM_T_P2, GM_KNN_P1, and GM_KNN_P2 for the line selection in genetic improvement (dataset: EYT_3) based on average Pearson's correlation (APC), Best20, mean arctangent absolute percentage error (MAAPE), and normalized root mean square error (NRMSE).

Dataset	Method	Metric	Mean	SE
EYT_3	C	APC	0.4737	0.0198
EYT_3	C	Best20	44.8718	2.0063
EYT_3	C	MAAPE	0.0276	0.0005
EYT_3	C	NRMSE	0.8816	0.0120
EYT_3	GM_KNN_P1	APC	0.3864	0.0207
EYT_3	GM_KNN_P1	Best20	43.2693	1.9854
EYT_3	GM_KNN_P1	MAAPE	0.0287	0.0005
EYT_3	GM_KNN_P1	NRMSE	0.9220	0.0103
EYT_3	GM_KNN_P2	APC	0.4951	0.0206
EYT_3	GM_KNN_P2	Best20	46.9872	1.9523
EYT_3	GM_KNN_P2	MAAPE	0.0269	0.0005
EYT_3	GM_KNN_P2	NRMSE	0.8698	0.0135
EYT_3	GM_T_P1	APC	0.3707	0.0149
EYT_3	GM_T_P1	Best20	37.1795	1.8791
EYT_3	GM_T_P1	MAAPE	0.0293	0.0004
EYT_3	GM_T_P1	NRMSE	0.9267	0.0068
EYT_3	GM_T_P2	APC	0.4880	0.0201
EYT_3	GM_T_P2	Best20	45.6410	2.1549
EYT_3	GM_T_P2	MAAPE	0.0272	0.0006
EYT_3	GM_T_P2	NRMSE	0.8735	0.0126

**TABLE A5:** Comparison of prediction performance between methods C, GM_T_P1, GM_T_P2, GM_KNN_P1, and GM_KNN_P2 for the line selection in genetic improvement (dataset: groundnut) based on average Pearson's correlation (APC), Best20, mean arctangent absolute percentage error (MAAPE), and normalized root mean square error (NRMSE).

Dataset	Method	Metric	Mean	SE
Groundnut	C	APC	0.6567	0.0172
Groundnut	C	Best20	53.8462	3.5912
Groundnut	C	MAAPE	0.1763	0.0052
Groundnut	C	NRMSE	0.7630	0.0164
Groundnut	GM_KNN_P1	APC	0.5271	0.0335
Groundnut	GM_KNN_P1	Best20	48.0769	4.1064
Groundnut	GM_KNN_P1	MAAPE	0.1934	0.0056
Groundnut	GM_KNN_P1	NRMSE	0.8549	0.0222
Groundnut	GM_KNN_P2	APC	0.6380	0.0176
Groundnut	GM_KNN_P2	Best20	54.4231	3.2009
Groundnut	GM_KNN_P2	MAAPE	0.1786	0.0050
Groundnut	GM_KNN_P2	NRMSE	0.7777	0.0165
Groundnut	GM_T_P1	APC	0.5487	0.0300
Groundnut	GM_T_P1	Best20	53.4615	3.4623
Groundnut	GM_T_P1	MAAPE	0.1924	0.0060
Groundnut	GM_T_P1	NRMSE	0.8460	0.0255
Groundnut	GM_T_P2	APC	0.6476	0.0182
Groundnut	GM_T_P2	Best20	55.1923	3.6588
Groundnut	GM_T_P2	MAAPE	0.1777	0.0051
Groundnut	GM_T_P2	NRMSE	0.7709	0.0175

**TABLE A6:** Comparison of prediction performance between methods C, GM_T_P1, GM_T_P2, GM_KNN_P1, and GM_KNN_P2 for the line selection in genetic improvement (Across_datasets) based on average Pearson's correlation (APC), Best20, mean arctangent absolute percentage error (MAAPE), and normalized root mean square error (NRMSE).

Dataset	Method	Metric	Mean	SE
Across_data	C	APC	0.482	0.020
Across_data	C	Best20	44.647	2.407
Across_data	C	MAAPE	0.113	0.002
Across_data	C	NRMSE	0.871	0.013
Across_data	GM_KNN_P1	APC	0.382	0.023
Across_data	GM_KNN_P1	Best20	39.041	2.541
Across_data	GM_KNN_P1	MAAPE	0.119	0.002
Across_data	GM_KNN_P1	NRMSE	0.919	0.012
Across_data	GM_KNN_P2	APC	0.481	0.020
Across_data	GM_KNN_P2	Best20	44.486	2.412
Across_data	GM_KNN_P2	MAAPE	0.114	0.002
Across_data	GM_KNN_P2	NRMSE	0.872	0.013
Across_data	GM_T_P1	APC	0.384	0.023
Across_data	GM_T_P1	Best20	39.408	2.392
Across_data	GM_T_P1	MAAPE	0.118	0.002
Across_data	GM_T_P1	NRMSE	0.919	0.012
Across_data	GM_T_P2	APC	0.485	0.020
Across_data	GM_T_P2	Best20	44.792	2.540
Across_data	GM_T_P2	MAAPE	0.114	0.002
Across_data	GM_T_P2	NRMSE	0.870	0.013

## References

[tpg220522-bib-0001] Battiston, F. , Nicosia, V. , & Latora, V. (2014). Structural measures for multiplex networks: A tutorial and comparative study. Physical Review E, 89, 032804. 10.1103/PhysRevE.89.032804 24730896

[tpg220522-bib-0002] Crossa, J. , Pérez‐Rodríguez, P. , Cuevas, J. , Montesinos‐López, O. , Jarquín, D. , de Los Campos, G. , Burgueño, J. , González‐Camacho, J. M. , Pérez‐Elizalde, S. , Beyene, Y. , Dreisigacker, S. , Singh, R. , Zhang, X. , Gowda, M. , Roorkiwal, M. , Rutkoski, J. , & Varshney, R. K. (2017). Genomic selection in plant breeding: Methods, models, and perspectives. Trends in Plant Science, 22(11), 961–975. 10.1016/j.tplants.2017.08.011 28965742

[tpg220522-bib-0003] Goyal, P. , & Ferrara, E. (2018). Graph embedding techniques, applications, and performance: A survey. Knowledge‐Based Systems, 151, 78–94. 10.1016/j.knosys.2018.03.022

[tpg220522-bib-0004] Haavelmo, T. (1943). The statistical implications of a system of simultaneous equations. Econometrica, 11, 1–12. 10.2307/1905714

[tpg220522-bib-0006] Montesinos‐López, O. A. , Montesinos‐López, A. , & Crossa, J. (2022). Multivariate statistical machine learning methods for genomic prediction. Springer. 36103587

[tpg220522-bib-0008] Pérez, P. , & De Los Campos, G. (2014). Genome‐wide regression and prediction with the BGLR statistical package. Genetics, 198(2), 483–495. 10.1534/genetics.114.164442 25009151 PMC4196607

[tpg220522-bib-0009] R Core Team . (2024). R: A language and environment for statistical computing . R Foundation for Statistical Computing. https://www.R‐project.org/

[tpg220522-bib-0010] United Nations, Department of Economic and Social Affairs, Population Division . (2019). World population prospects 2019: Highlights (ST/ESA/SER.A/423). https://population.un.org/wpp/Publications/Files/WPP2019_Highlights.pdf

[tpg220522-bib-0011] Vanraden, P. M. (2008). Efficient methods to compute genomic predictions. Journal of Dairy Science, 91, 4414–4423. 10.3168/jds.2007-0980 18946147

[tpg220522-bib-0012] VanRaden, P. M. (2017). Efficient methods to compute genomic predictions. Journal of Dairy Science, 100(12), 10363–10376. 10.3168/jds.2007-0980 18946147

[tpg220522-bib-0013] Wright, S. (1921). Correlation and causation. Journal of Agricultural Research, 201, 557–585.

