# Supplementary material for: A graph model for genomic prediction in the context of a linear mixed model framework
Source: Plant Genome. 2024 Oct 7;17(4):e20522. doi: 10.1002/tpg2.20522 (PMC11628911; doi:10.1002/tpg2.20522)
Supplement: Supplementary file 1 [file TPG2-17-e20522-s001.docx]

**Supplemental Material**

| **Table S1.** Comparison of prediction performance between methods C, GM_T_P1, GM_T_P2, GM_KNN_P1, GM_KNN_P2 for the Line Selection in Genetic Improvement (**Dataset: Indica**) based on APC, Best20, MAAPE and NRMSE | | | | |
| --- | --- | --- | --- | --- |
| Dataset | Method | Metric | Mean | SE |
| Indica | C | APC | 0.4852 | 0.0353 |
| Indica | C | Best20 | 41.1539 | 2.9795 |
| Indica | C | MAAPE | 0.1003 | 0.0038 |
| Indica | C | NRMSE | 0.8709 | 0.0235 |
| Indica | GM_KNN_P1 | APC | 0.4359 | 0.0277 |
| Indica | GM_KNN_P1 | Best20 | 36.1538 | 2.8789 |
| Indica | GM_KNN_P1 | MAAPE | 0.1019 | 0.0038 |
| Indica | GM_KNN_P1 | NRMSE | 0.8915 | 0.0182 |
| Indica | GM_KNN_P2 | APC | 0.4956 | 0.0329 |
| Indica | GM_KNN_P2 | Best20 | 40.0000 | 2.9832 |
| Indica | GM_KNN_P2 | MAAPE | 0.0995 | 0.0038 |
| Indica | GM_KNN_P2 | NRMSE | 0.8659 | 0.0230 |
| Indica | GM_T_P1 | APC | 0.4103 | 0.0281 |
| Indica | GM_T_P1 | Best20 | 31.1539 | 3.2294 |
| Indica | GM_T_P1 | MAAPE | 0.1037 | 0.0029 |
| Indica | GM_T_P1 | NRMSE | 0.9111 | 0.0223 |
| Indica | GM_T_P2 | APC | 0.4826 | 0.0331 |
| Indica | GM_T_P2 | Best20 | 37.8846 | 3.2387 |
| Indica | GM_T_P2 | MAAPE | 0.1001 | 0.0038 |
| Indica | GM_T_P2 | NRMSE | 0.8749 | 0.0237 |

***Figure S1.*** ***Indica*** *Dataset, comparison of prediction performance between the Conventional Method (C), GM_KNN_P1, GM_KNN_P2, GM_T_P1, and GM_T_P2 methods. The results are presented specifically for the GBLUP model, with a detailed focus on four key metrics: (A) APC, (B) Best20, (C) MAAPE, and (D) NRMSE.*

| **Table S2.** Comparison of prediction performance between methods C, GM_T_P1, GM_T_P2, GM_KNN_P1, GM_KNN_P2 for the Line Selection in Genetic Improvement (Dataset: **Japonica**) based on APC, Best20, MAAPE and NRMSE | | | | |
| --- | --- | --- | --- | --- |
| Dataset | Method | Metric | Mean | SE |
| Japonica | C | APC | 0.5553 | 0.0258 |
| Japonica | C | Best20 | 51.9231 | 2.4638 |
| Japonica | C | MAAPE | 0.1054 | 0.0031 |
| Japonica | C | NRMSE | 0.8396 | 0.0211 |
| Japonica | GM_KNN_P1 | APC | 0.4584 | 0.0298 |
| Japonica | GM_KNN_P1 | Best20 | 45.5769 | 3.7371 |
| Japonica | GM_KNN_P1 | MAAPE | 0.1101 | 0.0032 |
| Japonica | GM_KNN_P1 | NRMSE | 0.8958 | 0.0191 |
| Japonica | GM_KNN_P2 | APC | 0.5519 | 0.0263 |
| Japonica | GM_KNN_P2 | Best20 | 53.6538 | 2.5891 |
| Japonica | GM_KNN_P2 | MAAPE | 0.1059 | 0.0032 |
| Japonica | GM_KNN_P2 | NRMSE | 0.8409 | 0.0221 |
| Japonica | GM_T_P1 | APC | 0.4554 | 0.0359 |
| Japonica | GM_T_P1 | Best20 | 48.0769 | 3.4638 |
| Japonica | GM_T_P1 | MAAPE | 0.1103 | 0.0030 |
| Japonica | GM_T_P1 | NRMSE | 0.8936 | 0.0192 |
| Japonica | GM_T_P2 | APC | 0.5559 | 0.0275 |
| Japonica | GM_T_P2 | Best20 | 54.4231 | 3.0565 |
| Japonica | GM_T_P2 | MAAPE | 0.1059 | 0.0032 |
| Japonica | GM_T_P2 | NRMSE | 0.8366 | 0.0213 |

***Figure S2.*** ***Japonica*** *Dataset, comparison of prediction performance between the Conventional Method (C), GM_KNN_P1, GM_KNN_P2, GM_T_P1, and GM_T_P2 methods. The results are presented specifically for the GBLUP model, with a detailed focus on four key metrics: (A) APC, (B) Best20, (C) MAAPE, and (D) NRMSE.*

| **Table S3.** Comparison of prediction performance between methods C, GM_T_P1, GM_T_P2, GM_KNN_P1, GM_KNN_P2 for the Line Selection in Genetic Improvement (**Dataset: Maize**) based on APC, Best20, MAAPE and NRMSE. | | | | |
| --- | --- | --- | --- | --- |
| Dataset | Method | Metric | Mean | SE |
| Maize | C | APC | 0.4701 | 0.0141 |
| Maize | C | Best20 | 45.5172 | 1.8391 |
| Maize | C | MAAPE | 0.7367 | 0.0083 |
| Maize | C | NRMSE | 0.8887 | 0.0055 |
| Maize | GM_KNN_P1 | APC | 0.2684 | 0.0166 |
| Maize | GM_KNN_P1 | Best20 | 35.8621 | 2.0041 |
| Maize | GM_KNN_P1 | MAAPE | 0.7759 | 0.0058 |
| Maize | GM_KNN_P1 | NRMSE | 0.9675 | 0.0065 |
| Maize | GM_KNN_P2 | APC | 0.4433 | 0.0153 |
| Maize | GM_KNN_P2 | Best20 | 41.7241 | 2.4356 |
| Maize | GM_KNN_P2 | MAAPE | 0.7408 | 0.0078 |
| Maize | GM_KNN_P2 | NRMSE | 0.9004 | 0.007 |
| Maize | GM_T_P1 | APC | 0.3263 | 0.0167 |
| Maize | GM_T_P1 | Best20 | 42.069 | 1.8391 |
| Maize | GM_T_P1 | MAAPE | 0.7595 | 0.0074 |
| Maize | GM_T_P1 | NRMSE | 0.9481 | 0.0058 |
| Maize | GM_T_P2 | APC | 0.4647 | 0.0122 |
| Maize | GM_T_P2 | Best20 | 45.8621 | 1.9268 |
| Maize | GM_T_P2 | MAAPE | 0.7359 | 0.0079 |
| Maize | GM_T_P2 | NRMSE | 0.8909 | 0.0054 |

***Figure S3.*** *Maize Dataset, comparison of prediction performance between the Conventional Method (C), GM_KNN_P1, GM_KNN_P2, GM_T_P1, and GM_T_P2 methods. The results are presented specifically for the GBLUP model, with a detailed focus on four key metrics: (A) APC, (B) Best20, (C) MAAPE, and (D) NRMSE.*

| **Table S4.** Comparison of prediction performance between methods C, GM_T_P1, GM_T_P2, GM_KNN_P1, GM_KNN_P2 for the Line Selection in Genetic Improvement (Dataset: **Wheat_1**) based on APC, Best20, MAAPE and NRMSE. | | | | |
| --- | --- | --- | --- | --- |
| Dataset | Method | Metric | Mean | SE |
| Wheat_1 | C | APC | 0.5085 | 0.0117 |
| Wheat_1 | C | Best20 | 44.2308 | 2.1833 |
| Wheat_1 | C | MAAPE | 0.0429 | 0.0008 |
| Wheat_1 | C | NRMSE | 0.8616 | 0.0066 |
| Wheat_1 | GM_KNN_P1 | APC | 0.3352 | 0.0161 |
| Wheat_1 | GM_KNN_P1 | Best20 | 37.8846 | 1.5183 |
| Wheat_1 | GM_KNN_P1 | MAAPE | 0.0467 | 0.0007 |
| Wheat_1 | GM_KNN_P1 | NRMSE | 0.9422 | 0.0054 |
| Wheat_1 | GM_KNN_P2 | APC | 0.4985 | 0.0108 |
| Wheat_1 | GM_KNN_P2 | Best20 | 43.2692 | 1.8853 |
| Wheat_1 | GM_KNN_P2 | MAAPE | 0.0430 | 0.0008 |
| Wheat_1 | GM_KNN_P2 | NRMSE | 0.8665 | 0.0063 |
| Wheat_1 | GM_T_P1 | APC | 0.3548 | 0.0104 |
| Wheat_1 | GM_T_P1 | Best20 | 33.0769 | 1.2430 |
| Wheat_1 | GM_T_P1 | MAAPE | 0.0463 | 0.0007 |
| Wheat_1 | GM_T_P1 | NRMSE | 0.9360 | 0.0041 |
| Wheat_1 | GM_T_P2 | APC | 0.5119 | 0.0106 |
| Wheat_1 | GM_T_P2 | Best20 | 42.8846 | 2.2029 |
| Wheat_1 | GM_T_P2 | MAAPE | 0.0427 | 0.0008 |
| Wheat_1 | GM_T_P2 | NRMSE | 0.8590 | 0.0063 |

***Figure S4.*** ***Wheat_1*** *Dataset, comparison of prediction performance between the Conventional Method (C), GM_KNN_P1, GM_KNN_P2, GM_T_P1, and GM_T_P2 methods. The results are presented specifically for the GBLUP model, with a detailed focus on four key metrics: (A) APC, (B) Best20, (C) MAAPE, and (D) NRMSE.*

| **Table S5.** Comparison of prediction performance between methods C, GM_T_P1, GM_T_P2, GM_KNN_P1, GM_KNN_P2 for the Line Selection in Genetic Improvement (Dataset: **Wheat_2**) based on APC, Best20, MAAPE and NRMSE. | | | | |
| --- | --- | --- | --- | --- |
| Dataset | Method | Metric | Mean | SE |
| Wheat_2 | C | APC | 0.3831 | 0.0093 |
| Wheat_2 | C | Best20 | 40.0000 | 1.1034 |
| Wheat_2 | C | MAAPE | 0.0319 | 0.0005 |
| Wheat_2 | C | NRMSE | 0.9249 | 0.0041 |
| Wheat_2 | GM_KNN_P1 | APC | 0.1762 | 0.0129 |
| Wheat_2 | GM_KNN_P1 | Best20 | 31.5789 | 1.2267 |
| Wheat_2 | GM_KNN_P1 | MAAPE | 0.0335 | 0.0005 |
| Wheat_2 | GM_KNN_P1 | NRMSE | 0.9860 | 0.0029 |
| Wheat_2 | GM_KNN_P2 | APC | 0.3647 | 0.0100 |
| Wheat_2 | GM_KNN_P2 | Best20 | 40.1754 | 1.1534 |
| Wheat_2 | GM_KNN_P2 | MAAPE | 0.0321 | 0.0005 |
| Wheat_2 | GM_KNN_P2 | NRMSE | 0.9324 | 0.0042 |
| Wheat_2 | GM_T_P1 | APC | 0.1899 | 0.0178 |
| Wheat_2 | GM_T_P1 | Best20 | 31.9298 | 1.1928 |
| Wheat_2 | GM_T_P1 | MAAPE | 0.0337 | 0.0005 |
| Wheat_2 | GM_T_P1 | NRMSE | 0.9825 | 0.0030 |
| Wheat_2 | GM_T_P2 | APC | 0.3826 | 0.0089 |
| Wheat_2 | GM_T_P2 | Best20 | 41.4035 | 1.3387 |
| Wheat_2 | GM_T_P2 | MAAPE | 0.0319 | 0.0005 |
| Wheat_2 | GM_T_P2 | NRMSE | 0.9248 | 0.0038 |

***Figure S5. Wheat_2*** *Dataset, comparison of prediction performance between the Conventional Method (C), GM_KNN_P1, GM_KNN_P2, GM_T_P1, and GM_T_P2 methods. The results are presented specifically for the GBLUP model, with a detailed focus on four key metrics: (A) APC, (B) Best20, (C) MAAPE, and (D) NRMSE.*

| **Table S6.** Comparison of prediction performance between methods C, GM_T_P1, GM_T_P2, GM_KNN_P1, GM_KNN_P2 for the Line Selection in Genetic Improvement (Dataset: **Wheat_3**) based on APC, Best20, MAAPE and NRMSE. | | | | |
| --- | --- | --- | --- | --- |
| Dataset | Method | Metric | Mean | SE |
| Wheat_3 | C | APC | 0.3941 | 0.0161 |
| Wheat_3 | C | Best20 | 40.3509 | 2.0426 |
| Wheat_3 | C | MAAPE | 0.0317 | 0.0004 |
| Wheat_3 | C | NRMSE | 0.9194 | 0.0069 |
| Wheat_3 | GM_KNN_P1 | APC | 0.1779 | 0.0191 |
| Wheat_3 | GM_KNN_P1 | Best20 | 31.2281 | 1.3284 |
| Wheat_3 | GM_KNN_P1 | MAAPE | 0.0335 | 0.0004 |
| Wheat_3 | GM_KNN_P1 | NRMSE | 0.9852 | 0.0048 |
| Wheat_3 | GM_KNN_P2 | APC | 0.3800 | 0.0171 |
| Wheat_3 | GM_KNN_P2 | Best20 | 39.1228 | 1.7939 |
| Wheat_3 | GM_KNN_P2 | MAAPE | 0.0317 | 0.0004 |
| Wheat_3 | GM_KNN_P2 | NRMSE | 0.9246 | 0.0075 |
| Wheat_3 | GM_T_P1 | APC | 0.1662 | 0.0204 |
| Wheat_3 | GM_T_P1 | Best20 | 28.7719 | 1.5516 |
| Wheat_3 | GM_T_P1 | MAAPE | 0.0339 | 0.0005 |
| Wheat_3 | GM_T_P1 | NRMSE | 0.9868 | 0.0040 |
| Wheat_3 | GM_T_P2 | APC | 0.3911 | 0.0158 |
| Wheat_3 | GM_T_P2 | Best20 | 40.0000 | 1.8640 |
| Wheat_3 | GM_T_P2 | MAAPE | 0.0318 | 0.0004 |
| Wheat_3 | GM_T_P2 | NRMSE | 0.9205 | 0.0069 |

***Figure S6.*** ***Wheat_3*** *Dataset, comparison of prediction performance between the Conventional Method (C), GM_KNN_P1, GM_KNN_P2, GM_T_P1, and GM_T_P2 methods. The results are presented specifically for the GBLUP model, with a detailed focus on four key metrics: (A) APC, (B) Best20, (C) MAAPE, and (D) NRMSE.*

| **Table S7.** Comparison of prediction performance between methods C, GM_T_P1, GM_T_P2, GM_KNN_P1, GM_KNN_P2 for the Line Selection in Genetic Improvement (Dataset: **Wheat_4**) based on APC, Best20, MAAPE and NRMSE | | | | |
| --- | --- | --- | --- | --- |
| Dataset | Method | Metric | Mean | SE |
| Wheat_4 | C | APC | 0.3409 | 0.0103 |
| Wheat_4 | C | Best20 | 43.9286 | 1.1356 |
| Wheat_4 | C | MAAPE | 0.0321 | 0.0003 |
| Wheat_4 | C | NRMSE | 0.9412 | 0.0044 |
| Wheat_4 | GM_KNN_P1 | APC | 0.2439 | 0.0112 |
| Wheat_4 | GM_KNN_P1 | Best20 | 35.7143 | 1.8634 |
| Wheat_4 | GM_KNN_P1 | MAAPE | 0.0331 | 0.0002 |
| Wheat_4 | GM_KNN_P1 | NRMSE | 0.9722 | 0.0036 |
| Wheat_4 | GM_KNN_P2 | APC | 0.3422 | 0.0091 |
| Wheat_4 | GM_KNN_P2 | Best20 | 42.5000 | 1.8015 |
| Wheat_4 | GM_KNN_P2 | MAAPE | 0.0322 | 0.0003 |
| Wheat_4 | GM_KNN_P2 | NRMSE | 0.9414 | 0.0041 |
| Wheat_4 | GM_T_P1 | APC | 0.2027 | 0.0124 |
| Wheat_4 | GM_T_P1 | Best20 | 33.5714 | 2.1593 |
| Wheat_4 | GM_T_P1 | MAAPE | 0.0335 | 0.0002 |
| Wheat_4 | GM_T_P1 | NRMSE | 0.9797 | 0.0029 |
| Wheat_4 | GM_T_P2 | APC | 0.3361 | 0.0109 |
| Wheat_4 | GM_T_P2 | Best20 | 41.6071 | 1.5070 |
| Wheat_4 | GM_T_P2 | MAAPE | 0.0323 | 0.0003 |
| Wheat_4 | GM_T_P2 | NRMSE | 0.9431 | 0.0047 |

***Figure S7.*** ***Wheat_4*** *Dataset, comparison of prediction performance between the Conventional Method (C), GM_KNN_P1, GM_KNN_P2, GM_T_P1, and GM_T_P2 methods. The results are presented specifically for the GBLUP model, with a detailed focus on four key metrics: (A) APC, (B) Best20, (C) MAAPE, and (D) NRMSE.*

| **Table S8.** Comparison of prediction performance between methods C, GM_T_P1, GM_T_P2, GM_KNN_P1, GM_KNN_P2 for the Line Selection in Genetic Improvement (Dataset: **Wheat_5**) based on APC, Best20, MAAPE and NRMSE. | | | | |
| --- | --- | --- | --- | --- |
| Dataset | Method | Metric | Mean | SE |
| Wheat_5 | C | APC | 0.4484 | 0.0185 |
| Wheat_5 | C | Best20 | 44.6429 | 2.0620 |
| Wheat_5 | C | MAAPE | 0.0297 | 0.0003 |
| Wheat_5 | C | NRMSE | 0.8951 | 0.0093 |
| Wheat_5 | GM_KNN_P1 | APC | 0.3604 | 0.0207 |
| Wheat_5 | GM_KNN_P1 | Best20 | 41.7857 | 2.0133 |
| Wheat_5 | GM_KNN_P1 | MAAPE | 0.0307 | 0.0002 |
| Wheat_5 | GM_KNN_P1 | NRMSE | 0.9347 | 0.0086 |
| Wheat_5 | GM_KNN_P2 | APC | 0.4557 | 0.0173 |
| Wheat_5 | GM_KNN_P2 | Best20 | 44.4643 | 1.9643 |
| Wheat_5 | GM_KNN_P2 | MAAPE | 0.0293 | 0.0002 |
| Wheat_5 | GM_KNN_P2 | NRMSE | 0.8914 | 0.0092 |
| Wheat_5 | GM_T_P1 | APC | 0.3854 | 0.0223 |
| Wheat_5 | GM_T_P1 | Best20 | 46.7857 | 1.7817 |
| Wheat_5 | GM_T_P1 | MAAPE | 0.0305 | 0.0003 |
| Wheat_5 | GM_T_P1 | NRMSE | 0.9239 | 0.0088 |
| Wheat_5 | GM_T_P2 | APC | 0.4549 | 0.0185 |
| Wheat_5 | GM_T_P2 | Best20 | 43.9286 | 2.3084 |
| Wheat_5 | GM_T_P2 | MAAPE | 0.0295 | 0.0003 |
| Wheat_5 | GM_T_P2 | NRMSE | 0.8918 | 0.0096 |

***Figure S8.*** ***Wheat_5*** *Dataset, comparison of prediction performance between the Conventional Method (C), GM_KNN_P1, GM_KNN_P2, GM_T_P1, and GM_T_P2 methods. The results are presented specifically for the GBLUP model, with a detailed focus on four key metrics: (A) APC, (B) Best20, (C) MAAPE, and (D) NRMSE.*

| **Table S9.** Comparison of prediction performance between methods C, GM_T_P1, GM_T_P2, GM_KNN_P1, GM_KNN_P2 for the Line Selection in Genetic Improvement (Dataset: **Wheat_6**) based on APC, Best20, MAAPE and NRMSE. | | | | |
| --- | --- | --- | --- | --- |
| Dataset | Method | Metric | Mean | SE |
| Wheat_6 | C | APC | 0.5316 | 0.0094 |
| Wheat_6 | C | Best20 | 51.5686 | 0.9266 |
| Wheat_6 | C | MAAPE | 0.0342 | 0.0004 |
| Wheat_6 | C | NRMSE | 0.8472 | 0.0052 |
| Wheat_6 | GM_KNN_P1 | APC | 0.4174 | 0.0181 |
| Wheat_6 | GM_KNN_P1 | Best20 | 43.7255 | 1.7056 |
| Wheat_6 | GM_KNN_P1 | MAAPE | 0.0366 | 0.0004 |
| Wheat_6 | GM_KNN_P1 | NRMSE | 0.9094 | 0.0081 |
| Wheat_6 | GM_KNN_P2 | APC | 0.5369 | 0.0098 |
| Wheat_6 | GM_KNN_P2 | Best20 | 51.5686 | 1.0559 |
| Wheat_6 | GM_KNN_P2 | MAAPE | 0.0340 | 0.0004 |
| Wheat_6 | GM_KNN_P2 | NRMSE | 0.8436 | 0.0056 |
| Wheat_6 | GM_T_P1 | APC | 0.4591 | 0.0123 |
| Wheat_6 | GM_T_P1 | Best20 | 47.0588 | 1.2401 |
| Wheat_6 | GM_T_P1 | MAAPE | 0.0362 | 0.0004 |
| Wheat_6 | GM_T_P1 | NRMSE | 0.8897 | 0.0055 |
| Wheat_6 | GM_T_P2 | APC | 0.5334 | 0.0093 |
| Wheat_6 | GM_T_P2 | Best20 | 51.9608 | 0.9804 |
| Wheat_6 | GM_T_P2 | MAAPE | 0.0342 | 0.0004 |
| Wheat_6 | GM_T_P2 | NRMSE | 0.8461 | 0.0052 |

***Figure S9.*** ***Wheat_6*** *Dataset, comparison of prediction performance between the Conventional Method (C), GM_KNN_P1, GM_KNN_P2, GM_T_P1, and GM_T_P2 methods. The results are presented specifically for the GBLUP model, with a detailed focus on four key metrics: (A) APC, (B) Best20, (C) MAAPE, and (D) NRMSE.*
